# MicroRNA expression profiles of bovine milk exosomes in response to *Staphylococcus aureus* infection

**DOI:** 10.1186/s12864-015-2044-9

**Published:** 2015-10-16

**Authors:** Jiajie Sun, Kshama Aswath, Steven G. Schroeder, John D. Lippolis, Timothy A. Reinhardt, Tad S. Sonstegard

**Affiliations:** Animal Genomics and Improvement Laboratory, USDA-ARS, BARC-East, Beltsville, MD 20705 USA; School of Systems Biology, George Mason University, 10900 University Boulevard, Manassas, VA 20110 USA; Ruminant Diseases and Immunology Unit, National Animal Disease Center, USDA/ARS, Ames, IA 50010 USA; Acceligen Inc., 1246 University Avenue W, St. Paul, MN 55104 USA

**Keywords:** Bovine, Exosome, Milk, miRNA, Mastitis

## Abstract

**Background:**

Milk exosomes are a rich source of microRNAs (miRNAs) that are protected from degradation. Ingestion of milk and subsequent absorption of miRNAs into recipient cells by endocytosis may play a role in the regulation of neonatal innate and adaptive immunity. In contrast, the miRNA content of milk exosomes may also be indicative of a lactating animal’s health; whereby, the presence or absence of specific miRNAs could serve as biomarkers for early detection of bacterial infection that can lead to mastitis. In the present study, we therefore analyzed and compared miRNA expression profiles of milk exosomes from four Holstein cows obtained during mid-lactation prior to and after infection (48 h) of the mammary gland with *Staphylococcus aureus*.

**Methods:**

Milk exosomes, purified from control and *S. aureus* infected cows, were extracted for RNA. Following preparation indexed libraries from both groups the samples were subjected to next generation sequencing.

**Results:**

Next generation sequencing of eight, unpooled small RNA libraries derived from milk exosomes produced about 60.5 million high-quality, bovine-specific sequence reads for comparison of miRNA expression between treatments. Sequence identity analysis showed the miRNAs make up about 13 % of the average RNA content of these exosomes. Although 417 known bovine miRNAs were identified, miRNAs represented the least diverse class of RNA accounting for only 1 % of all unique sequences. The 20 most prevalent unique sequences within this class accounted for about 90 % of the total miRNA-associated reads across samples. Non-annotated, unique reads provided evidence for another 303 previously unknown bovine miRNAs. Expression analyses found 14 known bovine microRNAs significantly differed in frequency between exosomes from infected and control animals.

**Conclusions:**

Our survey of miRNA expression from uninfected milk exosomes and those produced in response to infection provides new and comprehensive information supporting a role for delivery into milk of specific miRNAs involved in immune response. In particular, bta-miR-142-5p, and −223 are potential biomarkers for early detection of bacterial infection of the mammary gland. Additionally, 22 mammary-expressed genes involved in regulation of host immune processes and response to inflammation were identified as potential binding targets of the differentially expressed miRNAs.

**Electronic supplementary material:**

The online version of this article (doi:10.1186/s12864-015-2044-9) contains supplementary material, which is available to authorized users.

## Background

MicroRNAs (miRNAs) are a class of endogenous non-coding small RNA molecules, typically 22 nucleotides in length, which bind primarily to the 3'UTR of target mRNAs to repress translation and/or accelerate the decay [1] of up to 30 % of all expressed transcripts [2]. Numerous studies have found roles for miRNAs in regulation of gene expression for important biological processes including cellular proliferation and differentiation, tissue development, and immune response. Of interest for our study, miRNAs help regulate the development of immune cells and modulate the innate and adaptive immune responses [3]. Some specific examples include the role of miR-150 in inhibiting synthesis of the transcription factor c-Myb to help regulate B-cell differentiation [4], and miR-126 targeting of insulin regulatory subunit-1 transcripts to positively control the fate of B-cells [5]. Similarly, miR-146a functions as a negative regulator of TNF receptor-associated factor 6 and Interleukin-1 receptor-associated kinase 1 transcripts during and/or after the innate immune system responds to a bacterial infection [6]. These examples demonstrate how microRNAs can exert some regulatory control of the immune system within a cell. More recent studies have shown that extracellular miRNAs stably present in body fluids through encapsulation into exosomes or microvesicles; may also play a significant role in intercellular communication during an immune response [7–9]. Therefore, the presence of specific miRNAs in exosomes may be indicative of various pathological conditions and provide biomarkers for detection of certain disease conditions [7].

Despite the recognized importance of miRNAs in host immune response to disease in humans and mice, their comparative roles in regulating the immune response of livestock are still in preliminary stages of study. Expression surveys of bovine miRNAs have been reported for embryos [10], muscle [11], adipose [12, 13], mammary gland [13], testicular and ovarian tissues [14–16], and various tissues involved in immune response [17]; but none of these reports have interrogated miRNA expression changes in response to infection. More recently, Lawless and colleagues reported two studies surveying miRNA expression changes in response to bacterial infection of a bovine cell line [18] and circulating monocytes from blood and milk [19]. In the latter report, bovine specific miRNAs were identified as amplifiers of monocyte inflammatory response networks and repressors of several metabolic pathways in response to *Staphylococcus uberis* (*S. uberis*) infection of the mammary gland.

Based on these findings and the hypothesis that exosomes may contain specific miRNAs indicative of infection means that characterizing the miRNA profile of bovine milk exosomes could provide further understanding of host immune response signaling during lactation. It is well known that human milk is the most essential nutritional source for infant health during postnatal development [20], containing nutrients that closely match infant requirements for brain development, growth, and a healthy immune system [21, 22]. In the past decade, many immune-related substances like secretory immunoglobulins, leukocytes, and antimicrobial factors such as lysozyme, lactoferrin, and oligosaccharides have been detected in milk [23]. More specific analyses of the exosome component of human milk found abundant immune-related proteins [24] as well as miRNAs [8, 9]. The presence of miRNAs has also been confirmed from cursory surveys of bovine [25–27] and porcine milk [28]. Taken together, the immune-related factors in milk exosomes could impart some pathogen resistance to the newborns lacking a fully developed immune system [29]. Some miRNAs may play a role in these effects [25, 26], since there is evidence that exosome miRNAs are readily absorbed within the neonate digestive tract after ingestion of milk [30].

Thus, the goal of our study was to comprehensively survey by deep sequencing the miRNA content of bovine milk exosomes derived from both healthy and infected mammary glands, and then attempt to identify if host response to infection significantly changes the content of specific miRNAs in exosomes. We chose to harvest exosomes from lactating Holstein cows challenged with *Staphylococcus aureus* (*S. aureus*); because Holsteins are the primary source of milk-related products consumed by humans globally, and *S. aureus* is a leading causative agent of bovine mastitis. Furthermore, identification of differentially expressed exosome miRNAs in this infection model serve as potential molecular targets for development of biomarkers assays to provide early detection of sub-clinical mastitis.

## Results and discussion

### Sequence analysis of milk exosome miRNAs

Next generation sequence analysis of the eight small RNA libraries derived from milk exosomes prior to (*N* = 4 control) and 48 h post *S. aureus* infection (*N* = 4 infected) yielded more than 76.4 million 36 nt sequences. After filtering, approximately 17.7 and 54.3 million high-quality sequence reads from the control and infected libraries were available for reference genome alignment, respectively (Additional file 1). The unbalanced read results between treatments was due to an intentional increase of sequencing coverage of the infection sample 2 library, which showed the rate of unique sequence discovery was reduced with additional depth of sequencing but still comparable to the other libraries with fewer sequence reads (Additional file 1 – last row). In total, all reads represented about 1.8 million unique sequences, and SOAP alignment [31] to the bosTau6 reference genome assembly [32] resulted filtered this set of unique sequences down to 747,169 bovine specific sequences corresponding to about 60.5 million total sequences (84 % of total) for further analyses. Consistent with a previous report [17], the relatively high sequence depth (avg. of 80 reads per unique sequence) and multiple, unpooled samples (*N* = 8) provided enough replication for bona fide discovery of potentially novel miRNAs expressed from the bovine genome.

Sequence identities were assigned to the 747,169 unique sequences by alignments to Rfam and bovine-specific sequences within mirBAse, Repeat and Reference mRNA databases. The average RNA content of milk exosomes based on alignments of mapped, annotated reads was approximately: 1) 58 % other non-coding RNA (mostly tRNA with some rRNA, snRNA and snoRNA – Additional file 1B), 2) 13 % known bovine miRNA, 3) 23 % repetitive sequences, 4) 1 % mRNA (unspliced and mature), and 5) 4 % non-annotated sequence (Additional file 1). Comparing the ratio of total sequence reads to unique sequences for each identity category revealed miRNA associated sequences were the least diverse class of RNA present in milk exosomes with mRNA and other non-coding RNA associated sequences having 500- and 50-fold more observed diversity, respectively. Because the purpose of this study was to characterize miRNA-associated sequences and attempts to identify bovine snoRNA and piRNA identities were hampered by lack of annotation for the bovine reference genome, none of the other categories of sequence were analyzed further. However, non-miRNA sequence data is available for future analyses through GEO accession GSE55144.

In order to examine the potential miRNA content of milk exosomes more deeply, the 4732 miRNA-associated unique sequences (<1 % of all unique RNA sequences) were clustered into homolog groups based on annotation of best match to miRBAse. Using SOAP and mirDeep2 [33] methods, identities of unique sequences could be assigned to 337 miRNA homolog groups corresponding to 376 miRNA loci and 411 miRNA homologs corresponding to 452 miRNA loci, respectively (Additional file 2A). The union of these two results yielded 417 known miRNAs corresponding to over 53 % of known bovine miRNAs in miRBase Release 19.

**Fig. 1:**
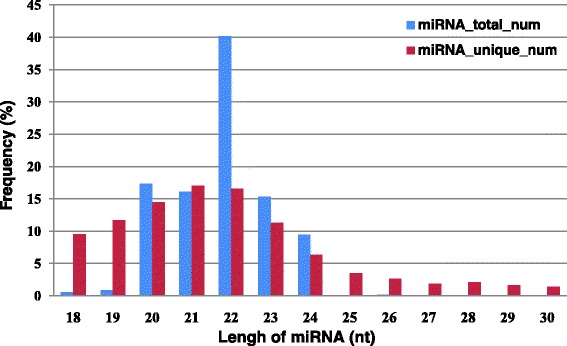
Distribution by length of mapped sequence reads. The distribution of mapped sequence reads from milk exosomes matching known bovine miRNAs found in miRBase binned by length (nt) and shown by percentage of unique (*red*) and total (*blue*) sequence read counts

The reduction of 4732 unique sequences to a set of 417 homologs is most likely attributable to the presence of isomiRs, which are heterogeneous variants of canonical miRNA species [34]. This finding was supported by variation in sequence read length (1–5 nt) for some unique sequence alignments within a homolog groups (Additional file 3). However, miRNA length distribution analysis (Fig. 1) showed the highest count of miRNA-associated reads was 22 nt in length (40.14 %), and the majority of unique sequences reads ranged within the expected length of 21–23 nt. Interestingly, a significant portion of unique reads was also present in the length ranging from 18–20 nt. This result requires further investigation, but the length variation may be attributed to enzyme modifications and imprecise processing of primary or precursor miRNAs by Drosha and Dicer enzymes, such as RNA editing in miRNA-mediated gene silencing [35], 3'-editing [36], and degradation of microRNAs by a family of exonucleases [37].

Looking at diversity within the 417 miRNA homolog groups, the number of total sequences representing isomiRs ranged from one to hundreds. As expected, the most highly expressed isomiR usually corresponded to the bovine reference sequence in miRBase (Additional file 3). A total of 201 miRNAs were found to have at least one isomiR expressed at a level of ≥10 sequence reads and more than 1432 different isomiRs were identified in total. The group designated bta-mir-2904 had the highest diversity of isomiRs at a level of ≥10 sequence reads with counts of 106 variants at the 5' end and 102 variants at the 3' end. The presence of such miRNAs variants was previously suggested to be cell type specific, have functional differences, and vary in their response to biological stimuli [38]. In our dataset, about 38 % of the isomiRs were variant at the 5' end, which should change the seed sequence (2–7 nt at the 5' end), and thereby effect changes in target mRNA and possibly miRNA function [39]. Overall, these results fully illustrated that miRNA diversity in exosomes is greatly extended through the presence of multiple isomiRs, which is a result that cannot be elucidated by real-time PCR or microarray expression analyses.

After accounting for isomiRs, we evaluated the overall distribution of miRNA diversity based on homolog groupings. The 15 most prevalent miRNAs accounted for between 76-88 % of the total normalized sequence reads depending on analysis method, while the members of this list were nearly conserved between methods (Additional file 2B). Furthermore, eight of these miRNAs (miR-30a-5p, −148a, 22-3p, −141, −186, −26a, −181a and -27b) shared common ranking as top 10 expressed miRNAs when parsing the sequence data based on treatment (control and infected exosomes), suggesting these miRNAs could be important nutritional components of milk. Recently, similar miRNAs were found by sequencing of lactating caprine mammary gland [40, 41], bovine mammary epithelial cells [18], and porcine [28] and human [9] milk. In the latter two studies; miR-30a, −148a, −141 and -27b were also represented as the top 10 miRNAs. Previous miRNA expression studies using bovine mammary tissue [42, 43] or milk [26] also found miR-148a and -181a as significantly elevated during lactation, respectively. However, most of the miRNAs reported as elevated in mammary tissue during ovine and bovine lactation do not match the most prevalent miRNAs from exosomes [44]. Some of these differences could be because previous studies only profiled expression for a pre-selected subset of miRNAs, which did not overlap with our Top 10 list. However, miR-148a-3p, which accounted for ~18.6 % of the total miRNA associated sequence reads, has previously been suggested as a nutritional biomarker corresponding to the protein content of various bovine-derived milk products [26].

Interestingly, feeding studies that have shown exogenous plant [45] or milk [30] miRNAs can be found in the sera and tissues and influence regulation of target genes in recipient animals. Combining the findings of these reports with the pleiotropic roles of the 10 most prevalent miRNAs in our study suggests the enrichment of specific miRNAs derived from mammary secretory cells may also influence development of the immune system of neonates. Specifically, the miR-30a-5p and -30d (members of the miR-30 family) are known to be involved in regulation of autophagy in cancer progression and treatment by suppressing the expression of *beclin 1* [46] and also cellular invasion and immunosuppression by targeting GalNAc transferase GALNT7 to increase synthesis of the immunosuppressive cytokine interleukin-10 [47]. Also widely studied for possible involvement in tumor progression, miR-148-3p has been shown to directly target the *TGIF2* gene in ovarian cancer [48], the drug metabolizing *PXR* gene in human cancer metastasis [49], and *CAND1* expression in human prostate cancer to promote prostate cell growth [50]. In the case of miR-27b, mRNA stability of PPARgamma is destabilized, which is often associated with chronic inflammatory diseases provoked by an immune response [51]. MiR-27b has also been found to be degraded by a viral transcript in lytic murine cytomegalovirus (MCMV) infection [52], further highlighting its role in immunity. Another prevalent milk miRNA, miR-181a, regulates T-cell selection by altering sensitivity to peptide antigens, which is partly achieved through the down regulation of multiple phosphatases that act as negative regulators of T cell receptor signaling [53]. Additional supporting evidence includes the roles of plasma miR-141 as a biomarker for colon cancers [54], miR-186 as a tumor-suppressor for the development and progression of lung adenocarcinoma [55], miR-26a in miRNA biogenesis to target *Lin28B* and *Zcchc11* as a suppression mechanism for tumor growth and metastasis [56], and miR-22 targeting of estrogen receptor α mRNA to inhibit estrogen signaling associated with some forms of breast cancer [57]. Finally, miR-191, a well characterized oncogenesis-related miRNA, is a biomarker of colorectal cancer [58], primary effusion lymphoma [59], and hepatocellular carcinoma [60]. Even though there is compelling evidence that the most prevalent miRNAs in our study could potentially exert an influence on immune response, the specific functional roles of each miRNA needs further detailed investigations to obtain a thorough understanding of the specific targets and mechanistic effects of consumption of miRNA-loaded bovine milk exosomes by a recipient animal. This is especially relevant considering that effects of microRNA consumption are still not well validated [61].

### Novel miRNA discovery

After assignment of sequence identities by various alignments (Additional file 1), nearly 38 % of all unique sequences had no identity or were considered non-annotated. A mireap analysis [62] of these sequences identified 562 potentially novel bovine miRNAs corresponding to 593 genomic loci (Additional file 4A). Interestingly, the total read counts for each of these potential miRNAs were much lower on average than for those matching known bovine miRs already present in miRBase. For instance, there were only 57 unique sequences with a total read count >100, and many of our initial novel miRNAs (N = 259) had read counts <10. Because known miRNAs use as little as 6–8 nucleotides on the 5' end to recognize target mRNAs [63, 64], the sequence of this seed region for the potentially novel miRNAs was compared to those seed regions found among 25,141 miRNAs in miRBase. A total 88.6 % of novel miRNAs had a seed sequence identical to those reported for known mature miRNAs, and this sequence conservation supports functionality for many of the miRNAs predicted by mireap analysis of non-annotated reads.

After removing the 259 unique sequences with <10 total reads counts, the remaining 303 unique sequences were also aligned to miRBase using BLAST. Only three unique sequences had 100 % identity with known miRNAs found exclusively in other species (eca-miR-1842, dre-miR-1388-5p, mmu-miR-219c-3p). In addition, 24 and 23 unique non-annotated sequences had significant homology with the bta-mir-2285 and bta-mir-2284 families, respectively (Additional file 4B). These results were consistent with a previous report that mir-2285 and −2284 families had possibly over 40 members spanning the entire bovine genome [65]. Although we are confident these analyses identified previously undiscovered bovine miRNAs, none of these sequences were used for the differential expression analyses between exosomes from control and infected milk.

### Differentially expressed miRNAs in response to *S. aureus* infection

**Fig. 2:**
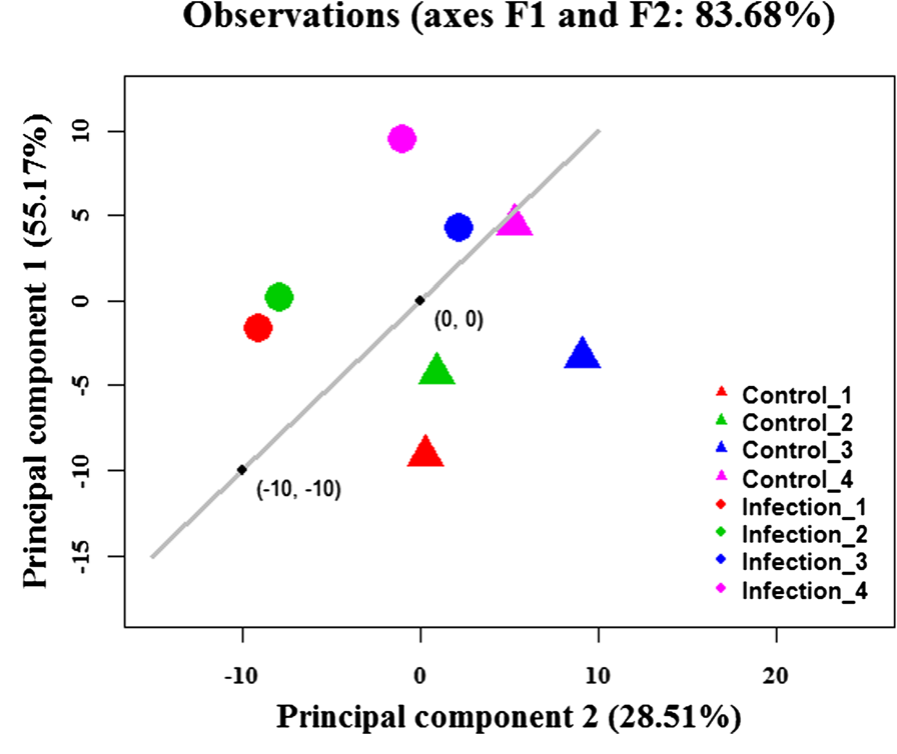
Principal component analysis of mapped reads by animal. Principal component analysis of the mapped sequence reads matching known bovine miRNAs from the eight individual libraries. Control represents sequences from non-infected milk exosome libraries and Infection represents those from infected quarters of the same animals 48 h post-infection

Compared to previous NGS studies investigating the milk miRNAs in bovine [26], porcine [28] and human [9], we generated sequence reads from unpooled miRNA libraries corresponding to individual animal replicates under different treatment (pre- and post-infection). This allowed exploration of statistically significant changes in miRNA content of milk exosomes in early response to *S. aureus* infection of the mammary gland. A separation of control (pre-infection) from infected samples was confirmed by principal component analysis (PCA) of the normalized sequence reads representing the 417 miRNA homolog clusters (Fig. 2). PC1 explained 55.17 % of the overall miRNA expression variability, whereas PC2 explained 28.51 % of the variability.

**Fig. 3:**
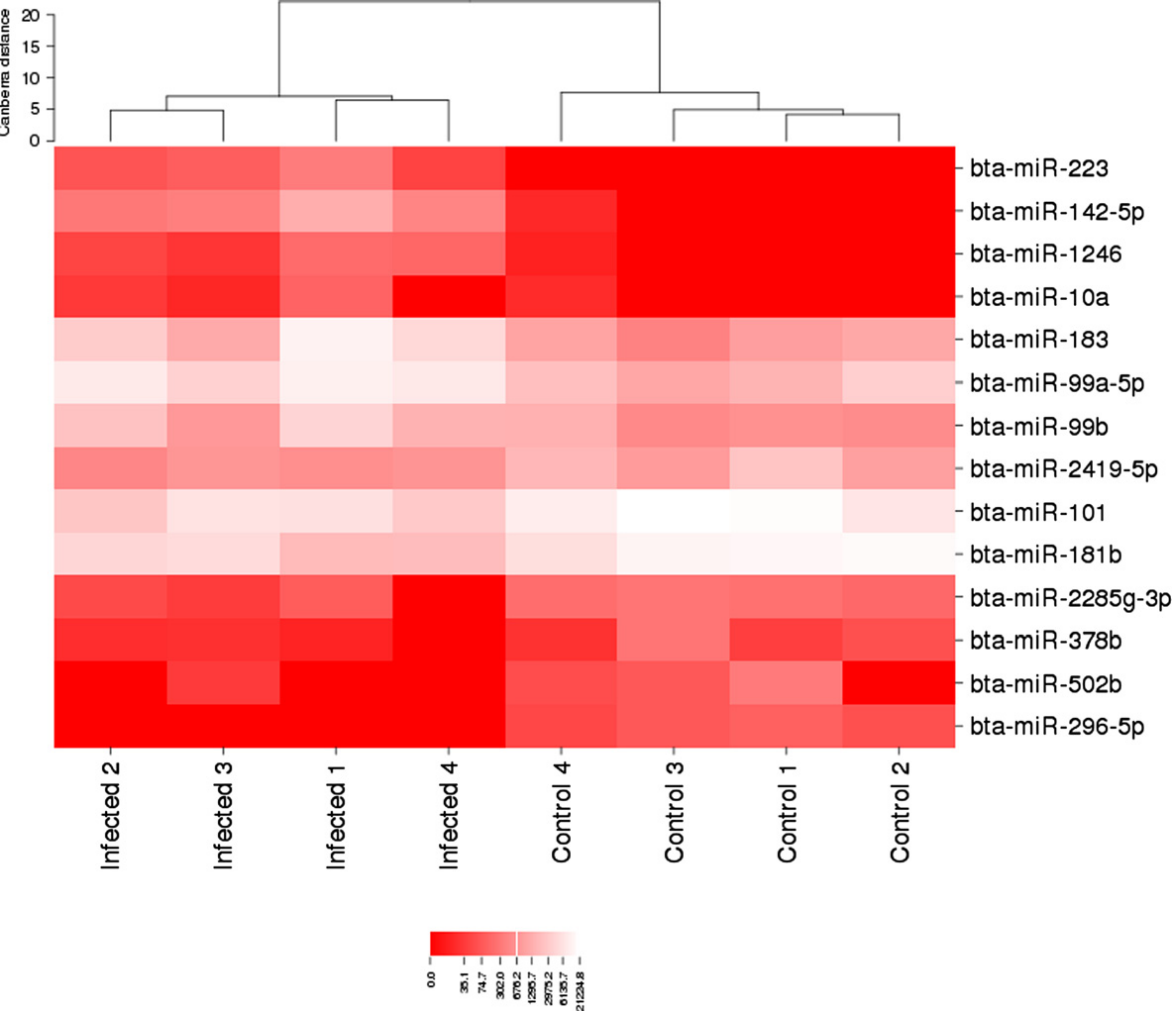
Heatmap of differentially expressed miRNA. Heatmap of 14 differentially expressed miRNAs (*P* < 0.05) between infected and non-infected as determined by miRDeep2 analysis of normalized sequence reads corresponding to known bovine miRNAs. Note: IN = Infected sample; C = Control or non-infected sample

The primary differential expression (DE) analysis was done using EdgeR and compared expression values of the unique miRNAs as determined by miRDeep2 normalization (Additional file 5). This approach detected a total of 14 DE miRNAs (Table 1). Heatmap analysis of these 14 DE microRNAs shows the clustering of infected from control samples (Fig. 3), ageing with the infection challenge statistics of these animals (Additional file 6).

**Table 1:** Known Bovine miRNAs with Significant Changes in Exosome Content in Response to Infection

miRNA name	*P* Value^a^	Change Relative to Control	Fold Change
bta-miR-142-5p	8.86E-08^c^	UP	263
bta-miR-296-5p	6.70E-05	DOWN	ND^b^
bta-miR-223	1.39E-04^c^	UP	ND^b^
bta-miR-1246	1.23E-03	UP	196
bta-miR-183	2.31E-03^c^	UP	6
bta-miR-502b	8.84E-03	DOWN	−13
bta-miR-378b	1.60E-02	DOWN	−10
bta-miR-2285 g-3p	1.73E-02^c^	DOWN	−4
bta-miR-99a-5p	1.93E-02^c^	UP	3
bta-miR-181b	2.25E-02	DOWN	−3
bta-miR-101	2.73E-02^c^	DOWN	−3
bta-miR-10a	2.87E-02	UP	15
bta-miR-99b	3.66E-02	UP	3
bta-miR-2419-5p	4.86E-02	DOWN	−3

^a^P Value Determined by EdgeR Analysis of Upper Quartile Normalized Sequence Reads corresponding to Bovine miRNA Homolog Groups

^b^bta-miR-296-5p was not present in infection samples and bta-miR-223 was not present in control samples

^c^Also significant by SOAP analysis

As a comparison to the EdgeR analysis, a differential expression analysis was also done by SOAP (Additional file 7), and six common miRNAs (bta-miR-101, −142-5p, −183, −2285 g-3p, −223 and -99a-5p) with differential presence in milk exosomes between uninfected and infected animals were identified (Table 1).

Comparing our DE miRNA results from milk to previously reported changes in bovine mammary-derived miRNA expression revealed observations in support of our findings. For example, we detected increases of bta-miR-142-5p and −223 in milk exosomes 48 h post-infection, while both lactating mammary epithelium [66] and bovine monocytes [19] under infection challenge with *S. uberis* also were found to increase levels of miR-223. Other reports surveying bovine milk [26, 27] found higher levels of miR-223 in colostrum possibly in support of increased immunity for early neonates or the mammary gland during a period of higher susceptibility (post-partum) to bacterial infection. Lawless and colleagues also found significant increases in miR-142-5p in mammary monocytes post-infection [19]. The cellular source of increases in our results, mammary epithelium or monocytes or both, remains to be determined; however, the lower levels of relative expression in comparison to the most prevalent miRNAs in exosomes suggests only a subset of cells responding to infection are contributing these RNAs to milk. The decreases in -15b, and -193a-5p found only using SOAP analysis are supported by similar findings in mammary epithelium [66] and monocytes [19], respectively. In contrast, we did not find any expression of bta-miR-2898, which was previously found to be upregulated in response to bacterial infection of mammary gland, where a potential role as a modulator of immune response was supported through differential regulation of A2M transcript binding [67].

Regarding the roles of these miRNAs in immune response, a previous study demonstrated elevated miR-142 in systemic lupus erythematosus patients compared to healthy controls [68]. MiR-142 has also been shown to be abundant in T cells [69], which implied its role as an immune-relevant miRNA while its function remains unclear. On the other hand, miR-223 has a potential role in balancing metabolism and immune response during infection [19], as previous bioinformatic analysis provides support for its importance in down-regulating lipid metabolism [66]; a process very important during milk production. Beyond its role in response to mammary infection in cattle, miR-223 was also recently reported to regulate granulopoiesis [70]. In spite of the limited knowledge of milk enriched miRNAs in response to bacterial infection, our results present significant information about highly expressed miRNAs in bovine milk exosomes from healthy and infected animals, and provides specific miRNAs that can be targeted for biomarker development as a potential means for early diagnostic detection of sub-clinical mastitis.

### Prediction and analysis of target genes

A total of 7961 unique genes were predicted by RNAhybrid to be targeted based by the six DE miRNAs found in common between both analysis methods (bta-miR-101, −142-5p, −183, −2285 g-3p, −223 and -99a-5p). Of these, 4511 genes were predicted to be targeted by up-regulated miRNAs; 1500 were targeted by down-regulated miRNAs; and 1950 were targeted by both up and down-regulated miRNAs (Additional file 8). Because many of these target mRNAs may not actually be expressed during lactation, we filtered our candidate list of 7961 genes for mammary specific genes by only selecting those encoding proteins identified in previous reports [71, 72]. From this reduced list of 2350 genes encoding mammary derived protein found in exosomes, a total of 219 overlapping target genes were identified (Additional file 9A). DAVID analysis results showed 11 Biological Processes GO terms for 22 genes, which were significantly related to host immune processes and inflammation (Additional file 9B). We therefore hypothesized that differentially expressed miRNAs can target sequences in these 22 genes to regulate the immune responses with *S. aureus* infection. This new knowledge of milk miRNA expression between healthy cows and cows with mastitis will provide information important for the immune function in the mammary gland.

## Conclusions

In conclusion, we have comprehensively analyzed the RNA content of milk exosomes from infected and uninfected animals using next generation sequencing to identify 417 known bovine miRNAs and 303 novel candidate miRNAs. Expression analyses identified six miRNAs that were significantly differentially present in exosomes in response to bacterial infection of the mammary gland, and provided two promising targets in bta-miR-142a and −223 for biomarker development of bacterial infection. Overall, this study expands the repertoire of bovine miRNAs and provides some specificity for the most prevalent miRNAs in milk, which may have effects on downstream gene expression through ingestion.

## Methods

### Milk exosomes collection

The United States Department of Agriculture (USDA), Agricultural Research Service (ARS), National Animal Disease Center (NADC) animal care and use committee approved all experimental animal procedures used in this study. No human subjects or data were used in this study. Milk exosomes were derived from Holstein cows (*N* = 4) housed at the NADC. Prior to infection all cows were healthy, and milk was confirmed free of bacteria. The exosome isolation methods and *S. aureus* challenge parameters were described previously for a proteome study of milk exosomes [72]. Briefly, control milk was aseptically collected from each quarter during mid-lactation (90 days in milk) and infected samples were collected 48 h post-infection from the matching quarter infused with approximately 400 colony forming units (CFU) of *S. aureus* (Newbolt strain) suspended in 10 ml of PBS. The crude exosomes were purified by sucrose gradient centrifugation and filtration [71]. The *S. aureus* infections were confirmed as previously described [72] and are summarized in Additional file 6.

### Small RNA library sequence analysis

Total RNA was extracted from pelleted exosomes using the manufacturer’s protocol for TRIzol® Reagent (Life Technologies, Carlsbad, CA, USA). Presence of post-extraction RNA was assessed by using a RNA 6000 pico assay run an Agilent Bioanalyzer (Agilent Technologies, Santa Clara, CA, USA). Eight Illumina-indexed libraries (four representing milk exosomes prior to infection and four from *S. aureus* infected samples 48 h post-infection) were constructed with an Illumina TruSeq small RNA library kit according to the manufacturers’ protocol (Illumina, Inc., San Diego, CA), which included size selection of the final library amplicons. All small RNA libraries were subjected to quality and purity check using Agilent’s 7500 DNA Bioanalyzer assay (Agilent Technologies, Santa Clara, CA, USA) and Qubit Fluorometric Quantitation (Life Technologies, Carlsbad, CA, USA). Quantitated libraries were diluted to 1 nM concentration and equal volumes were mixed for multiplex sequence analysis, and diluted to a final of 5 uM concentration for clustering on a Truseq flow-cell prior to analysis by single end sequencing (40 nt) on Illumina Genome Analyzer II.

Preliminary quality control analysis of the eight Fastq files generated using the FastQC package v0.10.0 [73] aimed to filter low quality short reads, remove poly A, trim 3'/5' adapter, and format into non-redundant Fasta files. Cutadapt v1.3 [74] was implemented to trim 3'/5' adaptor sequences, and reads less than 18 nt were removed from the dataset for analysis. The occurrence of each unique sequence read was tallied (Additional file 1). All unique sequence tags that passed through the above filtering criteria were mapped onto the bosTau6 version of the bovine reference genome assembly [32] using the SOAP algorithm [31] allowing up to 3 bp mismatches to detect isomiRs. Uniquely mapped tag sequences were aligned against Rfam v11.0, bovine miRNAs in miRBase v19.0, repeat database produced by RepeatMasker [75] and the coding genes of the reference genome [76]. Based on the annotation of the best matches, these unique sequences were classified as other non-coding RNA, known miRNA, genomic repeats or degradation products of mRNA. Sequences that mapped to the reference genome, but did not fit any of the above annotation categories were classified as ‘non-annotated’ reads.

A second method of data processing, named “miRDeep2-method” [29], was also used with some improvements. Under this method, Trimmed reads were then further filtered using Sickle [77] to remove low quality sequences with quality values <20. Reads that successfully passed filtering were aligned to the bovine miRBase v19.0 using miRDeep v2.0.0.5 with a Perl script “quantifier.pl”. All miRNAseq data was submitted to the NCBI Gene Expression Omnibus (GEO) database with the experiment series accession number GSE55144.

### miRNA expression analysis

To compare differentially expressed miRNAs between multiple samples through the miRDeep2-method, read count of each identified miRNA was normalized between libraries using upper-quartile normalization [78]. The R v3.0.2 Bioconductor package EdgeR v2.4.6 [79] was used to identify statistically differentially expressed miRNAs. Those miRNAs expressed at least once in control or infected samples were considered for further analysis. Differentially expressed miRNAs were defined as having a Benjamini and Hochberg corrected *P* value <0.05 [80]. The heat map of the differentially expressed miRNAs was generated using Cimminer [81] under the Canberra and Ward settings for Distance Method and Cluster Algorithm settings, respectively.

As a secondary validation analysis using SOAP [31], count data was individually normalized across 8 libraries using the formula: normalized expression (NE) = actual miRNA count/total count of clean reads × 10^6^ using SOAP. *P*-values were calculated from the normalized expression [82] in each replicate group compared between control and infected samples by:

$$ P\left(x\Big|y\right)={\left(\frac{N_{{}^2}}{N_1}\right)}^y\frac{\left(x+y\right)!}{x!y!{\left(1+\frac{N_2}{N_1}\right)}^{\left(x+y+1\right)}}\begin{array}{c}\hfill C\left(y\le {y}_{\min}\Big|x\right)={\displaystyle \sum_{y=0}^{y\le {y}_{\min }}p\left(y\Big|x\right)}\hfill \\ {}\hfill D\left(y\ge {y}_{man}\Big|x\right)={\displaystyle \sum_{y\ge {y}_{\max}}^{\infty }p\left(y\Big|x\right)}\hfill \end{array} $$

where x and y represent normalized expression levels, and the N1 and N2 represented total sequence read count for a given miRNA from the infected and control small RNA libraries, respectively. A specific miRNA was deemed to be significantly differentially expressed when the *P*-value was <0.01, and there was at least a 2-fold change in normalized sequence counts across all four replicates. The significant differentially expressed miRNAs identified by both SOAP and miRDeep2 approaches were selected for further target prediction analysis.

### Novel miRNA prediction

To investigate discovery of potentially novel miRNAs, all unmapped reads from the “SOAP-method” were run through Mireap [62], a prediction software that explores secondary structure characteristic, the Dicer cleavage site and the minimum free energy of input sequence reads. The following criteria [83] were used for identifying potentially novel miRNAs from unique sequence reads: 1) aligned to non-annotated regions of a bovine reference genome, 2) folded into hairpin secondary structures (with flanking sequence added) and unique sequence read was present in one arm of miRNA precursor, 3) demonstrated a 2 nt 3'-overhang between the sequence read (presumed miRNA mature strand) and its complementary strand (miRNA*), 4) folded hairpin miRNA precursors lacked large internal loops or bulges, 5) had a free energy of hybridization ≤ −18 kcal/mol as a folded hairpin structure, 6) carried a higher minimal free energy indexes (MFEIs) as a predicted precursor miRNA than other different types of RNAs.

### Target predictions

Differentially expressed miRNAs from the exosomes were further analyzed to predict potential regulatory target transcripts. *Bos taurus* mRNA sequences were downloaded from the NCBI database and input into RNAhybrid software [84] based on a series of rules: 1) no mismatch present between base pairs 1–9 on the 5' end of the miRNA/target RNA duplex, 2) G:U pairing was permitted for up to 3 bases. In addition, we also used Reinhardt et al.’s data [27] gained by analysis of bovine milk exosome proteome infected with *S. aureus* to narrow down the pool of potential targets predicted by computational analysis. Only target sites which were identified by the two approaches were further considered. Using DAVID Bioinformatics Resources 6.7 [85] only predicted targets which were identified by both computational and experimental analysis from infected exosomes were submitted to David Gene Ontology (GO) terms.

## Additional files

Additional file 1:
**Summary (A) of Unique and Total Sequence Reads Counts and Summary (B) of Sequence Identities for Matches to Rfam.** (XLSX 17 kb) Additional file 2:
**Total (A) and Normalized (B) Sequence Counts per Known Bovine miRNA Homolog Group.** (XLSX 73 kb) Additional file 3:
**Analysis Results for Potential Bovine IsomiRs from Milk Exosomes.** (XLSX 259 kb) Additional file 4:
**Identification of Novel Bovine miRNAs Based on Seed Sequence Alignments (A) and Sequence Identity of Novel Bovine miRNAs from BLAST Analysis of miRBase 19 (B).** (XLSX 112 kb) Additional file 5:
**Full Results of EdgeR Differential Expression Analysis of Normalized Sequence Reads Corresponding to Known Bovine miRNAs by miRDeep.** (XLSX 33 kb) Additional file 6:
**Animal Infection and Milk Exosome Data from Staph infected quarters.** Control exosomes came from same quarter prior to infection. (XLSX 9 kb) Additional file 7:
**Full Results of** **SOAP-based** **Differential Expression Analysis of Normalized Sequence Reads Corresponding to Known Bovine miRNAs (Animal/Replicate 1–4).** (XLSX 60 kb) Additional file 8:
**Predicted mRNA Targets of Differentially Expressed miRNAs in Milk Exosomes.** (XLSX 2115 kb) Additional file 9:
**A) List of 219 Genes Encoding Proteins Differentially Expressed (DE) in Milk Exosomes and Predicted Targets of DE miRNAs and B) Gene Ontology Results of DAVID Analysis of 219 mRNA Targets Expressed in Mammary Gland.** (XLSX 35 kb)
